# Effects of Topical Prostaglandin Analog on Macular Thickness Following Cataract Surgery with Postoperative Topical Bromfenac Treatment

**DOI:** 10.3390/jcm9092883

**Published:** 2020-09-07

**Authors:** Kee Sup Park, Kyoung Nam Kim, Kyeung Min Kim, Han Min Lee, Sung Bok Lee, Nam Ho Lee, Chang-Sik Kim

**Affiliations:** 1 Department of Ophthalmology, Chungnam National University College of Medicine, Daejeon 35015, Korea; red-mirr@hanmail.net (K.S.P.); atomico123@naver.com (K.M.K.); lihanil12@naver.com (H.M.L.); sblee@cnu.ac.kr (S.B.L.); kcs61@cnu.ac.kr (C.-S.K.); 2 Department of Ophthalmology, Chungnam National University Hospital, 282 Munhwa-ro, Jung-gu, Daejeon 35015, Korea; 3 Mindeulle Eye Clinic, 9 Samsannam-ro, Boeun 28950, Korea; 74amg@naver.com

**Keywords:** bromfenac, cystoid macular edema, prostaglandin analog, phacoemulsification, glaucoma

## Abstract

Purpose: To evaluate changes in macular thickness in patients continuing prostaglandin analog (PGA) treatment during the perioperative period involving bromfenac treatment. Methods: Patients with glaucoma who were using a topical PGA were randomly assigned to two groups in this randomized controlled trial: PGA continuing study group and PGA discontinued glaucoma control group. Patients without ocular diseases other than cataract were enrolled into the non-glaucomatous group. After the cataract surgery, the patients used bromfenac twice per day for 4 weeks. Optical coherence tomography was performed in all patients preoperatively and at 1 month postoperatively. Changes in macular thickness were compared among the three groups. Results: There were 32 eyes in the study group, 33 eyes in the glaucoma control group, and 58 eyes in the non-glaucomatous group. We found statistically significant postoperative changes in central macular thickness in all groups (4.30 ± 8.01 μm in the PGA continuing group, 9.20 ± 13.88 μm in the PGA discontinued group, and 7.06 ± 7.02 μm in the non-glaucomatous group, all *p* < 0.008), but no significant difference among the three groups (*p* = 0.161). Cystoid macular edema occurred in only one patient in the non-glaucomatous group (*p* = 0.568). Conclusions: Continuous use of PGAs during the perioperative period was not significantly associated with increased macular thickness after uncomplicated cataract surgery. In the absence of other risk factors (e.g., capsular rupture, uveitis, or diabetic retinopathy), discontinuing PGAs for the prevention of macular edema after cataract surgery with postoperative bromfenac treatment is unnecessary in patients with glaucoma.

## 1. Introduction

Pseudophakic cystoid macular edema (CME), Irvine–Gass syndrome, is one of the most common causes of vision loss after cataract surgery [1,2,3,4]. CME is characterized by thickening of the central retina, due to accumulation of fluid in the outer plexiform layer and abnormal perifoveal retinal capillary permeability. It has a peak incidence at approximately 4–5 weeks postoperatively [5,6,7,8]. The incidence of CME is reportedly between 0.1% and 7.0%; the disparity mainly results from differences in diagnostic techniques, such as clinical examinations, fluorescein angiography, and optical coherence tomography (OCT) [1,2,9,10,11]. Spectral domain OCT (SD-OCT) is a safe and noninvasive objective imaging method for the quantification of macular edema [5,12]. Subclinical CME (i.e., without clinical symptoms and not detected by slit-lamp biomicroscopy), which does not result in visual impairment and is characterized by perifoveal edema and cystic spaces on SD-OCT, has been described following uncomplicated cataract surgery in several studies [6,13].

Risk factors for pseudophakic CME have been reported to include intraoperative complications (e.g., posterior capsule rupture or vitreous loss), diabetes mellitus or preoperative diabetic retinopathy, previous retinal vein occlusion, epiretinal membrane, preoperative use of topical prostaglandin analogs (PGAs), and postoperative use of PGAs and β-blockers [1,10,14,15,16,17]. Because PGAs are powerful ocular hypotensive agents, effectively preserving visual function with few systemic adverse effects, they are commonly used as first-line agents in medical treatment of glaucoma [18,19,20,21]. Although PGAs are associated with a risk of CME due to enhanced vascular incompatibility caused by the proinflammatory effect [22], the perioperative use of PGAs in cataract surgery remains controversial; their effects on pseudophakic CME are unclear. Henderson et al. reported that the preoperative use of PGAs was predictive of pseudophakic CME development [1]. In contrast, Chu et al. reported that use of PGAs did not increase the risk of pseudophakic CME [9]. Additionally, two recent prospective studies showed contrary results to each other with regard to the relationship between PGAs and pseudophakic CME [10,23]. PGA use is becoming more prevalent as a treatment for glaucoma, while aging of the population is resulting in greater numbers of people who require concurrent glaucoma therapy and cataract surgery; thus, it is necessary to determine whether PGAs should be discontinued to prevent pseudophakic CME.

Topical ophthalmic nonsteroidal anti-inflammatory drugs (NSAIDs) are commonly used as treatment and prophylaxis for postoperative inflammation and pseudophakic CME in cataract surgery. To the best of our knowledge, there have been no studies evaluating the risk of macular edema in patients continuously treated with PGAs after cataract surgery during the postoperative NSAID treatment period. Therefore, we analyzed the postoperative changes in macular thickness between patients with glaucoma who were continuing PGAs and patients with glaucoma who were discontinuing PGAs, during the period of cataract surgery involving postoperative bromfenac treatment. In addition, we compared the macular thickness in these patients with glaucoma to that in normal controls.

## 2. Methods

### 2.1. Patients

The study protocol for this randomized controlled trial was approved by the Institutional Review Board of Chungnam National University Hospital, Republic of Korea (IRB number: 2019-01-071); the study was conducted in accordance with all relevant requirements of the Declaration of Helsinki. Patients from among those with planned cataract surgery (i.e., phacoemulsification with posterior chamber intraocular lens insertion) in the glaucoma clinic of Chungnam National University Hospital between January 2019 and June 2020, were consecutively enrolled in the study if they satisfied the relevant inclusion criteria.

Patients diagnosed with glaucoma (primary open-angle, primary angle-closure, normal-tension, or pseudoexfoliation), who were using a topical PGA as monotherapy for intraocular pressure control for at least 6 months before cataract surgery, were randomly assigned to one of two glaucoma groups, according to the order of scheduled surgery: odd-numbered patients were assigned to the PGA-continuing group and continued to use a PGA throughout the perioperative period, while even-numbered patients were assigned to the PGA discontinued glaucoma (control) group and their usage of PGA was discontinued from 1 day before to 1 month after the cataract surgery. If both eyes met the above inclusion criteria, the first operated eye was included in the study. Informed consent was acquired from all patients in the two glaucoma groups.

The non-glaucomatous group consisted of patients without ocular diseases (other than cataract), who were matched (at a 2:1 ratio) with patients in the PGA continuing group in terms of age and history of diabetes mellitus. These patients were retrospectively recruited; the requirement for informed consents was waived for the retrospective review of existing patient records for the non-glaucomatous group only. If both eyes met the inclusion criteria, the first operated eye was included in the study. Patients were excluded if they had ocular disease (e.g., uveitis), retinopathy (e.g., retinal vein occlusion, diabetic retinopathy, epiretinal membrane, or age-related macular degeneration), optic neuropathy other than glaucoma, a history of previous intraocular surgery, high myopia (axial length > 26 mm), and/or any artifacts in preoperative macular SD-OCT; patients were also excluded if they required topical anti-glaucoma medication for postoperative elevated intraocular pressure, except patients in the study group with PGA monotherapy.

In our clinic, all patients with planned cataract surgery underwent thorough ophthalmological examinations, including best-corrected visual acuity, autorefractometry, slit-lamp biomicroscopy, Goldmann applanation tonometry, dilated fundus examination, fundus photography, IOLMaster (Carl Zeiss, Jena, Germany), and SD-OCT (Cirrus HD OCT; Carl Zeiss Meditec, Dublin, CA, USA).

All cataract surgeries were performed after topical anesthesia with 0.5% proparacaine hydrochloride eye drops (Alcaine; Alcon, Inc., Fort Worth, TX, USA) by a single experienced surgeon (K.N.K.). A clear 2.2-mm corneal incision was made on the temporal side; the surgery was performed using identical viscoelastic material (Hyalu inj. 1%; Hanmi Pharm, Seoul, Korea) and the Centurion Vision System apparatus (Alcon, Inc., Fort Worth). A foldable intraocular lens was inserted in the bag. All patients were instructed to use 0.1% fluorometholone (Fumeron; Hanlim Pharm, Seoul, Korea), 1.5% levofloxacin (Cravit; Santen, Osaka, Japan), and diquafosol (Diquas-s; Santen) four times per day, and 0.1% bromfenac sodium hydrate (Bronuck; Taejoon Pharm, Seoul, Korea) twice per day, for 4 weeks postoperatively. Postoperative routine follow-ups were scheduled at 1 and 4 days postoperatively, and at 1 month after surgery. At 1 month postoperatively, thorough ophthalmic examinations were performed; these included best-corrected visual acuity, autorefractometry, slit-lamp biomicroscopy, Goldmann applanation tonometry, dilated fundus examination, fundus photography, and SD-OCT.

### 2.2. Macular Thickness Measurement by SD-OCT

**Figure 1:**
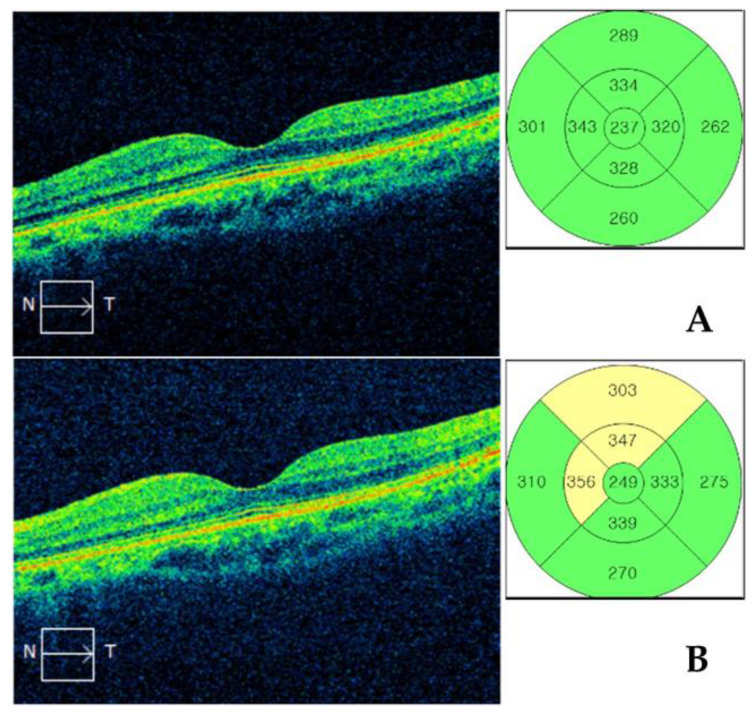
Spectral domain optical coherence tomography macular cube scan. Nine macular thickness values corresponding to the Early Treatment of Diabetic Retinopathy Study (ETDRS) areas were used in the analyses. The three concentric circles in 6 × 6-mm scans had diameters of 1, 3, and 6 mm; each ring (inner and outer) was divided into four quadrants (superior, nasal, inferior, and temporal). (**A**) Preoperative and (**B**) postoperative images of the same representative patient.

Macular cube 512 × 128 scans were performed using Cirrus HD OCT through a fully dilated pupil. Preoperative SD-OCT imaging was performed in all patients within 2 weeks preoperatively; the examination was repeated at 1 month postoperatively. For inclusion, SD-OCT images were required to have a signal strength ≥6 and to be free of artifacts caused by eye motion, blinking, misalignment, or segmentation error. Macular thickness measurements corresponding to the Early Treatment of Diabetic Retinopathy Study (ETDRS) areas were used in the analyses. The diameters of the three concentric circles in 6 × 6-mm scans were 1, 3, and 6 mm, respectively; each ring (inner and outer rings) was divided into four quadrants (superior, nasal, inferior, and temporal, Figure 1). Significant pseudophakic macular edema was defined as (1) macular edema with cystic changes in the outer plexiform and inner nuclear layers [24]; and/or (2) an increase in central macular thickness (CMT) ≥ 20% or 50 μm, compared with the preoperative value [23].

### 2.3. Statistical Analysis

PASW software, version 18.0 (SPSS Inc., Chicago, IL, USA) was used for all statistical analyses. One-way analysis of variance (ANOVA) was used to compare preoperative and postoperative macular thicknesses measured by SD-OCT in the three groups. The degrees of postoperative change in macular thickness, based on preoperative measurement, were also compared among the three groups by using ANOVA. The paired *t*-test was used to compare preoperative and postoperative macular thicknesses in each group. The incidences of significant pseudophakic macular edema were compared among the three groups. In all analyses, *p* < 0.05 was considered to indicate statistical significance.

## 3. Results

### 3.1. Demographics

Data from 113 eyes of 113 patients who underwent routine phacoemulsification were analyzed. There were no definite intraoperative complications, such as posterior capsule rupture, iris prolapse, or prolonged surgery time. The demographic characteristics of the patients enrolled in the study are presented in Table 1. There were 32 eyes in the PGA continuing group, 33 in the PGA discontinued group, and 58 eyes in the non-glaucomatous group; the mean patient ages were 67.8 ± 12.1 years, 67.7 ± 9.5 years, and 68.8 ± 8.8 years (*p* = 0.865), respectively. There were no significant differences in sex or prevalence of systemic disease, nor in preoperative best-corrected visual acuity, intraocular pressure, central corneal thickness, or axial length, among the three groups. Average retinal nerve fiber layer thicknesses were significantly thinner in the PGA continuing and PGA discontinued groups than those in the non-glaucomatous group (73.20 ± 13.96 μm and 69.00 ± 11.38 μm vs. 89.22 ± 10.03 μm, respectively, *p* < 0.001).

### 3.2. Comparisons of Macular Thickness Measured by SD-OCT

Table 2 shows comparisons of preoperative macular thickness among the PGA continuing, PGA discontinued, and non-glaucomatous groups. Preoperative CMT did not significantly differ among the three groups (249.73 ± 19.89 μm, 257.65 ± 17.88 μm, and 250.20 ± 22.58 μm, respectively, ANOVA, *p* = 0.348). The macular thicknesses in other ETDRS areas (except CMT, inner nasal, and outer nasal areas) were significantly thinner in the PGA continuing and PGA discontinued groups than those in the non-glaucomatous group (all *p* < 0.032).

Table 3 shows comparisons of postoperative macular thickness among the PGA continuing, PGA discontinued, and non-glaucomatous groups. Postoperative CMT did not significantly differ among the three groups (254.03 ± 20.42 μm, 263.85 ± 22.75 μm, and 257.26 ± 22.69 μm, respectively, *p* = 0.126). Macular thicknesses in other ETDRS areas (except CMT, inner nasal, and outer nasal areas) were significantly thinner in the PGA continuing and PGA discontinued groups than those in the non-glaucomatous group (ANOVA, all *p* < 0.030).

Table 4 shows a comparison of postoperative changes in macular thickness among the three groups. Postoperative changes in CMT did not significantly differ among the PGA continuing, PGA discontinued, and non-glaucomatous groups (4.30 ± 8.01 μm, 6.20 ± 10.88 μm, and 7.06 ± 7.02 μm, respectively, *p* = 0.161). Macular thicknesses in all of the ETDRS areas, including CMT, showed significant thickening in all three groups postoperatively (all *p* < 0.041). However, the degrees of macular thickening in all of the ETDRS areas did not significantly differ among the three groups (all *p* > 0.120). One patient in the control group (1.72%) exhibited significant pseudophakic macular edema (CME with low-intensity cystic spaces detected on OCT). By contrast, no patients exhibited significant pseudophakic macular edema in either the PGA continuing or PGA discontinued groups.

Linear regression analyses revealed that continuation of PGAs was not associated with a postoperative CMT increase (β = 1.195, 95% CI −0.879 to 3.269, *p* = 0.256). No clinical factors, including type of glaucoma and type of PGA (i.e., latanoprost, tafluprost, and bimatoprost), were significantly associated with a postoperative CMT increase (all *p* > 0.131, Table 5).

## 4. Discussion

In this study, we analyzed the macular thickness in patients with glaucoma who were continuing PGA treatment, then compared it with the macular thickness in patients with glaucoma who had discontinued PGA treatment, as well as in normal control patients, to evaluate the effects of perioperative PGA use on macular edema after cataract surgery. Conventional phacoemulsification was performed and 0.1% bromfenac sodium hydrate treatment was administered postoperatively. The results of our study indicate that the macular thicknesses of all ETDRS areas, including the CMT, were significantly increased after cataract surgery in all three groups. However, the postoperative increase in macular thickness did not significantly differ in any of the ETDRS areas among the three groups. In addition, the incidence of significant macular edema did not significantly differ among the three groups. These results suggest that continuous use of PGAs perioperatively did not significantly affect macular thickness after cataract surgery during the application of bromfenac treatment.

Many risk factors have been suggested to contribute to pseudophakic CME, including intraoperative complications (e.g., posterior capsule rupture and vitreous loss), diabetes mellitus or preoperative diabetic retinopathy, previous retinal vein occlusion, and epiretinal membrane [14,15,17]. The perioperative use of topical PGAs has also been suggested as a risk factor for pseudophakic CME [1,10,12,16,25,26,27,28]. The physiologically active prostaglandins include PGD2, PGE2, PGF2α, PGI2, and thromboxane A2; among these prostaglandins, PGF2α has been shown to have ocular hypotensive effects in humans. PGAs used in glaucoma therapy (e.g., latanoprost, tafluprost, travoprost, and bimatoprost) are structurally related to prostaglandin PGF2α [29]. PGAs relax ciliary muscles [30,31] and enhance the levels of matrix metalloproteinases (MMPs) in ciliary muscles and sclera. These MMPs induce the decomposition of collagen, resulting in reconstitution of the extracellular matrix, which increases uveoscleral outflow [32,33,34]. The reported side effects of PGAs include conjunctival hyperemia, ocular pruritus, periorbital skin pigmentation, iris color change, periorbital fat atrophy, hypertrichosis, intraocular inflammation, herpes simplex keratitis reactivation, and macular edema [35,36,37,38].

Since the introduction of PGAs, numerous patients with PGA-associated CME have been described [28,37,39,40,41,42]. Nevertheless, there is no consensus with regard to PGA use in patients undergoing cataract surgery; it is unclear whether PGAs are associated with an elevated risk of pseudophakic CME [43]. Henderson et al. [1,5] reported that, when patients with diabetes mellitus were excluded, preoperative use of PGAs was a significant risk factor for pseudophakic CME (*p* = 0.04). In their prospective cohort study, Lee et al. [10] reported an odds ratio of 5.51 for risk of pseudophakic CME in the primary open-angle glaucoma group using PGAs. Holló et al. [44] reported that when inflammation is present in the eye, the early use of PGA after cataract surgery has the potential to increase the risk of CME. In contrast, Chu et al. [9] conducted a database study of 81,984 eyes and found that the incidence of CME was 1.17% in patients without intraoperative complications, diabetes, or other risk factors (e.g., epiretinal membrane, uveitis, retinal vein occlusion, or retinal detachment repair). In that study, use of PGAs did not lead to an elevated risk of CME. In their prospective randomized clinical trial, Fakhraie et al. [23] concluded that administration of a single PGA (latanoprost) after cataract surgery had no measurable effect on macular thickness. Accordingly, there are no guidelines for the perioperative use of PGAs, and surgeons have approached use of these drugs on an individual basis [16]. In a survey of ophthalmologists in the UK, 40.3% of ophthalmologists reported that they stopped using PGAs after cataract surgery; approximately 50% reported that they routinely stopped using PGAs in all patients [43]. In a survey performed in Greece, 80% of responders reported that they discontinued PGAs preoperatively. Of those who stopped PGA use, 65% stopped using PGAs postoperatively, while 33% also stopped using these drugs preoperatively [45].

NSAIDs block conversion of arachidonic acid to prostaglandins by inhibiting cyclooxygenase-1 and -2 (COX-1 and COX-2) [46,47]. Naturally occurring prostaglandins play key roles in proinflammatory reactions, stimulating pain, vasodilation, blood–ocular barrier disruption, and leukocyte migration. Therefore, the use of NSAIDs results in anti-inflammatory effects. Cataract surgery has progressed markedly over the past several decades. Despite surgical improvements, however, pseudophakic CME continues to be a common cause of postoperative visual disturbance [2,48,49]. After general surgical trauma, including routine cataract surgery on the iris, ciliary body, or lens epithelial cells, prostaglandin levels increase in the aqueous humor. Prostaglandins can diffuse into the vitreous humor, reach the retina, and disrupt the blood–retinal barrier [50]. Topical NSAIDs are increasingly used in ophthalmology; their Food and Drug Administration-approved indications include the management of post-cataract surgery pain and inflammation (nepafenac, bromfenac) or post-cataract surgery inflammation (diclofenac) [51]. Because of their ability to inhibit COX, other indications have been explored, including prevention and treatment of pseudophakic CME. There is evidence that perioperative use of NSAIDs, alone or in combination with topical corticosteroids, reduces the likelihood of pseudophakic CME [4,52,53,54,55,56]. Modjtahedi et al. [57]. conducted a retrospective review of 89,731 patients; they concluded that the use of topical NSAIDs reduced the incidence of pseudophakic CME, compared with that in patients who did not receive topical NSAIDs (1.3% vs. 1.7%, respectively, *p* < 0.001). To the best of our knowledge, among the previous studies regarding the relationship between PGAs and pseudophakic CME, no studies have used NSAIDs routinely after cataract surgery.

The most common NSAID ophthalmic solutions used in South Korea are 0.1% diclofenac sodium, 0.45% ketorolac tromethamine, 0.1% pranoprofen, and 0.1% bromfenac [58]. Bromfenac was used in our study, twice per day for 4 weeks, as a brominated NSAID with potent in vitro anti-inflammatory effects [59]. We encountered only one patient (1.72%) in the non-glaucomatous group with pseudophakic CME, which constitutes an incidence similar to that in the previous study.

In this study, preoperative macular thicknesses were thinner in the study groups (PGA continuing and PGA discontinued groups) than those in the non-glaucomatous group, except for the central area and the inner and outer nasal areas. This is because the patients in the two study groups had glaucoma and a thinner peripapillary retinal nerve fiber layer, compared to patients in the non-glaucomatous group (average retinal nerve fiber layer (RNFL) thicknesses were 73.20 ± 13.96 μm in the PGA continuing group, 69.00 ± 11.38 μm in the PGA discontinued group, and 89.22 ± 10.03 μm in non-glaucomatous group, *p* < 0.001). Notably, retinal ganglion cells and the retinal nerve fiber layer over the macula comprise 30–35% of the thickness of the macula [60]; thus, the loss of retinal ganglion cells and RNFL has been proposed to reflect a reduction in macular thickness. In addition, Greenfield et al. [61] demonstrated that macular thickness in patients with glaucoma was significantly lower compared to that in non-glaucomatous patients due to the existing correlation between macular thickness and RNFL thickness in glaucoma. Macular thickness of the nasal area showed no difference among the three groups in the present study. This is presumably because the papillomacular bundle of RNFL tends to be preserved until the terminal stage in patients with glaucoma [62]. There were no significant differences in preoperative CMT among the three groups in the present study. This was likely because the central area contains the minimal number of ganglion cells and retinal nerve fibers [63], resulting in no significant difference between patients with glaucoma and normal controls.

Our study had some limitations. First, the postoperative SD-OCT imaging was performed only once, at 1 month postoperatively. Although it may be insufficient to assess the long-term effects of PGAs after cataract surgery, previous studies reported the highest incidence of significant macular edema at 4 weeks, when using OCT after cataract surgery [6,8]. Second, our participants were routinely treated with a topical NSAID (0.1% bromfenac). Therefore, the results of this study indicating that PGAs did not affect pseudophakic CME may be applicable only when NSAIDs are used after cataract surgery. Third, to eliminate possible confounding factors, patients with ophthalmic diseases other than glaucoma and cataracts were excluded. Therefore, it is difficult to generalize the results of this study to patients with known risk factors of pseudophakic CME, such as uveitis, diabetic retinopathy, retinal vein occlusion, and epiretinal membrane. Finally, the study included a relatively small number of patients treated with PGAs. Therefore, to confirm the results of this study, further research involving a larger cohort of patients is necessary.

In conclusion, continuous PGA use in the perioperative period was not significantly associated with increased macular thickness after uncomplicated cataract surgery. In the absence of other risk factors (e.g., uveitis or diabetic retinopathy) and in an environment where postoperative prophylactic use of NSAIDs is permitted, it is unnecessary to discontinue use of PGAs to prevent macular edema after cataract surgery in patients with glaucoma.

## Figures and Tables

**Table 1:** Patient demographic and clinical characteristics.

Characteristics	PGA Continuing (A)	PGA Discontinued (B)	Non-Glaucomatous (C)	*p*-Value
No. of patients (no. of eyes)	32 (32)	33 (33)	58 (58)	
Age (years)	67.8 ± 12.1	67.7 ± 9.5	68.8 ± 8.8	0.865 *
Sex (male/female)	7/25	10/23	18/40	0.630 ^†^
Systemic disease, n (%)				
DM	9 (28.1)	9 (27.3)	17 (29.3)	0.978 ^†^
HTN	7 (21.9)	11 (33.3)	17 (29.3)	0.581 ^†^
CVA	3 (9.4)	4 (12.1)	5 (8.6)	0.861 ^†^
Type of glaucoma, n (%)				0.557 ^‡^
Normal-tension	19 (59.4%)	17 (51.5%)	N/A	
Primary open-angle	8 (25.0%)	7 (21.2%)	N/A	
Pseudoexfoliation	4 (12.5%)	5 (15.2%)	N/A	
Primary angle-closure	1 (3.1%)	4 (12.1%)	N/A	
Best-corrected visual acuity (logMAR)				
Preoperative	0.41 ± 0.24	0.25 ± 0.21	0.33 ± 0.27	0.069 *
Postoperative	0.09 ± 0.18	0.06 ± 0.13	0.04 ± 0.08	0.186 *
Intraocular pressure (mmHg)				
Preoperative	18.47 ± 3.28	16.70 ± 2.74	17.12 ± 3.36	0.106 *
Postoperative	17.37 ± 2.44	16.50 ± 1.67	16.64 ± 3.37	0.460 *
Central corneal thickness (μm)	533.40 ± 42.51	532.95 ± 37.01	548.44 ± 35.59	0.141 *
Axial length (mm)	23.27 ± 0.99	23.92 ± 1.25	23.39 ± 1.07	0.100 *
Average RNFL thickness (μm)	73.20 ± 13.96	69.00 ± 11.38	89.22 ± 10.03	<0.001 * (A = B < C)
Type of PGA (n)				0.441 ^‡^
Latanoprost 0.005%	15	16	N/A	
Tafluprost 0.0015%	12	15	N/A	
Bimatoprost 0.03%	5	2	N/A	

Values are means ± standard deviations. CVA, cardiovascular attack; DM, diabetes mellitus; HTN, hypertension; PGA, prostaglandin analog; RNFL, retinal nerve fiber layer. * *p*-value from one-way analysis of variance (Bonferroni’s post hoc test). ^†^ *p*-value from chi-squared test. ^‡^ *p*-value for the comparison between the PGA continuing and PGA discontinued groups.

**Table 2:** Comparison of preoperative macular thicknesses among the PGA continuing, PGA discontinued, and non-glaucomatous groups.

ETDRS Areas	PGA Continuing (A)	PGA Discontinued (B)	Non-Glaucomatous (C)	*p*-Value *
Central	249.73 ± 19.89	257.65 ± 17.88	250.20 ± 22.58	0.348 (A = B = C)
Inner superior	309.43 ± 14.85	309.05 ± 15.95	316.46 ± 11.34	0.032 (A = B < C)
Inner temporal	290.00 ± 17.46	295.35 ± 18.23	307.16 ± 16.24	0.001 (A = B < C)
Inner inferior	294.93 ± 19.19	301.00 ± 17.22	314.06 ± 12.02	0.001 (A = B < C)
Inner nasal	310.20 ± 16.73	315.70 ± 14.99	316.90 ± 13.43	0.143 (A = B = C)
Outer superior	267.83 ± 17.17	258.30 ± 16.22	276.10 ± 11.23	0.001 (A = B < C)
Outer temporal	248.77 ± 16.59	245.45 ± 10.86	264.94 ± 13.57	0.001 (A = B < C)
Outer inferior	241.77 ± 18.46	240.90 ± 13.71	260.14 ± 12.25	0.001 (A = B < C)
Outer nasal	281.63 ± 19.42	278.25 ± 15.74	288.02 ± 15.14	0.057 (A = B = C)

Values are means ± standard deviations (μm). ETDRS, Early Treatment of Diabetic Retinopathy Study; PGA, prostaglandin analog. * *p*-value from one-way analysis of variance (Bonferroni’s post hoc test).

**Table 3:** Comparison of postoperative macular thicknesses among the PGA continuing, PGA discontinued, and non-glaucomatous groups.

ETDRS Areas	PGA Continuing (A)	PGA Discontinued (B)	Non-Glaucomatous (C)	*p*-Value *
Central	254.03 ± 20.42	263.85 ± 22.75	257.26 ± 22.69	0.126 (A = B = C)
Inner superior	317.70 ± 15.29	316.10 ± 11.61	324.86 ± 12.24	0.030 (A = B < C)
Inner temporal	299.17 ± 15.37	306.20 ± 14.97	316.68 ± 13.77	0.001 (A = B < C)
Inner inferior	303.07 ± 17.88	308.00 ± 13.65	321.80 ± 12.02	0.002 (A = B < C)
Inner nasal	318.93 ± 15.98	321.70 ± 15.61	325.28 ± 12.42	0.581 (A = B = C)
Outer superior	274.13 ± 16.16	266.20 ± 11.92	281.70 ± 11.63	0.001 (A = B < C)
Outer temporal	255.87 ± 16.07	249.55 ± 12.20	271.24 ± 14.95	0.001 (A = B < C)
Outer inferior	247.73 ± 17.59	245.10 ± 9.30	266.56 ± 13.17	0.001 (A = B < C)
Outer nasal	288.37 ± 15.41	283.75 ± 12.57	294.33 ± 17.60	0.087 (A = B = C)

Values are means ± standard deviations. ETDRS, Early Treatment of Diabetic Retinopathy Study; PGA, prostaglandin analog. * *p*-value from one-way analysis of variance (Bonferroni’s post hoc test).

**Table 4:** Comparison of postoperative changes in macular thickness among the PGA continuing, PGA discontinued, and non-glaucomatous groups.

ETDRS Areas	PGA Continuing (A)	*p*-Value	PGA Discontinued (B)	*p*-Value	Non-Glaucomatous (C)	*p*-Value	*p*-Value *
Central	4.30 ± 8.01	0.001	6.20 ± 10.88	0.008	7.06 ± 7.02	0.006	0.161 (A = B = C)
Inner superior	8.27 ± 9.33	0.001	7.05 ± 10.58	0.001	8.40 ± 6.48	0.001	0.909 (A = B = C)
Inner temporal	9.16 ± 12.51	0.001	10.85 ± 13.87	0.008	9.52 ± 10.59	0.002	0.465 (A = B = C)
Inner inferior	8.13 ± 9.65	0.001	7.00 ± 11.69	0.012	7.74 ± 6.41	0.001	0.942 (A = B = C)
Inner nasal	8.73 ± 15.78	0.001	6.00 ± 14.97	0.030	8.38 ± 10.63	0.002	0.541 (A = B = C)
Outer superior	6.30 ± 8.07	0.001	7.90 ± 9.27	0.004	5.60 ± 6.36	0.004	0.632 (A = B = C)
Outer temporal	7.10 ± 9.25	0.001	4.10 ± 16.35	0.008	6.30 ± 10.31	0.001	0.147 (A = B = C)
Outer inferior	5.97 ± 10.92	0.001	4.20 ± 11.61	0.041	6.42 ± 7.42	0.001	0.120 (A = B = C)
Outer nasal	6.73 ± 13.38	0.002	5.50 ± 12.84	0.026	6.31 ± 13.16	0.001	0.772 (A = B = C)

Values are means ± standard deviations. ETDRS, Early Treatment of Diabetic Retinopathy Study; PGA, prostaglandin analog. * *p*-value from one-way analysis of variance (Bonferroni’s post hoc test).

**Table 5:** Clinical factors associated with postoperative central macular thickness (μm) increases.

Clinical Factors	r	β	95% Confidence Interval	*p*-Value
Age (years)	0.066	0.060	−0.123 to 0.244	0.515
Sex (male)	0.071	1.317	−2.389 to 5.022	0.482
Systemic disease				
DM	0.086	−1.693	−5.972 to 2.586	0.434
HTN	0.161	−2.895	−6.664 to 0.874	0.131
CVA	0.008	−0.224	−6.682 to 6.234	0.945
Type of glaucoma, n *				
Normal-tension	0.250	5.456	−0.682 to 11.595	0.080
Primary open-angle	0.093	−2.431	−9.951 to 5.089	0.519
Pseudoexfoliation	0.090	−2.794	−11.775 to 6.186	0.535
Primary angle-closure	0.225	−10.206	−23.043 to 2.632	0.117
Preoperative BCVA (mmHg)	0.057	2.015	−5.015 to 9.045	0.571
Preoperative IOP (mmHg)	0.014	−0.039	−0.572 to 0.495	0.886
CCT (μm)	0.055	−0.013	−0.061 to 0.034	0.586
Axial length (mm)	0.140	1.133	−0.470 to 2.735	0.164
Average RNFL thickness (um)	0.073	0.046	−0.080 to 0.171	0.472
PGA continuing	0.115	1.195	−0.879 to 3.269	0.256
Type of PGA *				
Latanoprost 0.005%	0.218	4.708	−1.403 to 10.820	0.128
Tafluprost 0.0015%	0.005	−1.438	−7.870 to 4.994	0.655
Bimatoprost 0.03%	0.224	−6.947	−15.736 to 1.842	0.119

r, correlation coefficient; β, regression coefficient; DM, diabetes mellitus; HTN, hypertension; CVA, cardiovascular attack; BCVA, best-corrected visual acuity; IOP, intraocular pressure; CCT, central corneal thickness; RNFL, retinal nerve fiber layer; PGA, prostaglandin analog. * Analysis performed in patients with glaucoma but not in the non-glaucomatous group.
